# High Complement Factor I Activity in the Plasma of Children with Autism Spectrum Disorders

**DOI:** 10.1155/2012/868576

**Published:** 2011-10-24

**Authors:** Naghi Momeni, Lars Brudin, Fatemeh Behnia, Berit Nordström, Ali Yosefi-Oudarji, Bengt Sivberg, Mohammad T. Joghataei, Bengt L. Persson

**Affiliations:** ^1^School of Natural Sciences, Linnaeus University, 39182 Kalmar, Sweden; ^2^Department of Clinical Physiology, Kalmar County Hospital, 39185 Kalmar, Sweden; ^3^Department of Occupational Therapy, University of Social Welfare and Rehabilitation Sciences, Tehran, Iran; ^4^Department of Health Sciences, Autism Research, Faculty of Medicine, Lund University, Box 157, 22100 Lund, Sweden; ^5^Cellular and Molecular Research Centre, Tehran University of Medical Sciences (TUMS), Tehran, Iran

## Abstract

Autism spectrum disorders (ASDs) are neurodevelopmental and behavioural syndromes affecting social orientation, behaviour, and communication that can be classified as developmental disorders. ASD is also associated with immune system abnormality. Immune system abnormalities may be caused partly by complement system factor I deficiency. Complement factor I is a serine protease present in human plasma that is involved in the degradation of complement protein C3b, which is a major opsonin of the complement system. Deficiency in factor I activity is associated with an increased incidence of infections in humans. In this paper, we show that the mean level of factor I activity in the ASD group is significantly higher than in the control group of typically developed and healthy children, suggesting that high activity of complement factor I might have an impact on the development of ASD.

## 1. Introduction

Autism spectrum disorders (ASDs) are characterized by impairments in social interaction, communication, and repetitive or restricted patterns of interests, or behaviours, and are classified as developmental disorders in DSM-IV [1]. ASD is about 4-5 times more prevalent in boys than in girls. The ratio is estimated to range from 5.5 : 1.4 to 16.8 : 4.0 [2]. Recent research clearly indicates that the underlying causes of autism are neurobiological disorders and combinations of different factors, such as environmental and genetic factors, and abnormality in the communication between neurons, probably associated with an abnormal set of neuropeptides in the brain [3–9]. 

The symptoms of ASD have been linked with elevated plasma levels of serotonin [10, 11] and opioid [12], abnormal levels of melatonin [13], altered levels of activity of the serine protease prolyl endopeptidase [14], and infectious and immunological factors such as abnormalities of T cells, B cells, natural killer (NK) cells, and of the complement system [15–21]. 

The complement system comprises a group of proteins which, when activated, provide one of the first lines of defence by promoting lysis and the removal of invading microbes. Activation of the complement system in response to an infection or foreign antigen is achieved via three complement pathways, the classical pathway, which is activated by antigen-antibody complexes, the lectin pathway, which is activated by the interaction of microbial carbohydrates with mannose-binding proteins in the plasma and tissue fluids, and the alternative complement pathway, which is activated by C3b binding to microbial surfaces and to antibody molecules. All of the three pathways converge with the activation of the central C3 component. This leads to a final common pathway with assembly of the C5–C9 components to form a cell surface transmembrane pore (membrane attack complex) [22, 23]. It has been shown by comparison with healthy control children that several differentially expressed proteins are related to the complement system in children with ASD [22]. The alternative pathway consists of six proteins: C3, factor B, factor D, factor H, factor I, and properdin. The plasma glycoprotein factor I (C3b/C4b inactivator) is a serine protease that acts as a regulator of the complement C3 cascade. Factor I has a molecular weight of about 88 kDa, consists of two disulfide-linked polypeptide chains (50 kDa and 38 kDa, respectively), and is synthesized as a single-chain precursor in the liver [24, 25]. Factor I cleaves C3b and C4b in a reaction, where fI is dependent on various cofactors, such as factor H, C4b-binding protein CR1 and membrane cofactor protein (MCP) [26]. Factor I-mediated cleavage of the *α* chain of C3b liberates 3 fragments with molecular weights of 68 kDa, 43 kDa, and 2 kDa. Degradation of C3b by fI abrogates the action of this protein in the C3 pathway [27]. Complement C3b is the major opsonin of the complement system which facilitates the phagocytosis process by coating antigens (each of the phagocytes expresses a complement receptor such as CR1, CR3, or CR4 that binds C3b, C4b, or C3bi) [28, 29]. Factor I deficiency can be conferred by a C3 deficiency, since this also increases susceptibility to pyogenic infections by Neisseria meningitides, Haemophilus influenza, and Streptococcus pneumonia and increases the incidence of immune complex diseases due to impaired complement-mediated function [30]. Immune system abnormalities have been associated with autism [15–20], and it has been suggested that children with ASD might have an increased incidence of bacterial inflammation [31]. Immunological aspects of the early onset of autism have recently highlighted the fact that immune dysfunction may occur in some children with autism [31, 32].

Having previously discovered altered levels of the serine protease prolyl endopeptidase in children with ASD [14], the aim of this study was to investigate if an association exists between serine protease fI deficiency and the development of ASD.

## 2. Materials and Methods

### 2.1. Participants

Thirty children with ASD and thirty typical control children participated in this study. The ASD group comprised 23 boys and 7 girls with a mean age of 4.5 years (age range 3–9 years). The control group comprised 13 boys and 17 girls, mean age 6.0 years (age range 3–12 years), (Table 1). 

Children in the ASD group were recruited from the Autism Rehabilitation Centre at the University of Social Welfare and Rehabilitation Sciences in Tehran, Iran. After having obtained informed consent from the parents, blood samples were collected. All children with ASD were examined by clinical specialists on autism. A child psychiatrist and a child neurologist or child psychologist examined all of the children. All consultants agreed on the diagnosis of autism according to the DSM-IV criteria [1]. However, diagnostic procedures applied in Europe and in the US/Canada using the autism diagnostic observation schedule (ADOS) [33] and the Autism Diagnostic Interview—Revised [34] were not used in the diagnostic process applied in Iran. This shortcoming was compensated for by the extensive clinical experience by the child neurologist/child psychiatrist who was familiar with the core behaviours in autism stated by the American Academy of Pediatrics in its Embargo from 2007 [35]. The control group consisted of typically developed and healthy children showing no signs of neurodevelopmental disorders who were recruited from the same area as the children with ASD. Children who had any kind of infection/infectious disease within two weeks prior to the time of examination were excluded from this study.

The study was approved (MT/1247) by the ethics committee of the Iran University of Medical Sciences, Tehran.

### 2.2. Procedure

#### 2.2.1. Blood Sample Collection

Blood samples were collected by a paediatric nurse, and those from the children diagnosed with autism were collected under the supervision of a child psychiatrist with special training in the field of childhood psychosis. Venous blood was collected into 3 mL EDTA tubes (Vacutainer System; Becton-Dickinson Inc., Plymouth, UK), and plasma was separated immediately thereafter by centrifugation at 1,300 g for 10 min at 4°C. Thereafter, an inhibitors cocktail (30 *μ*L per 1 mL plasma) was added to the resultant plasma sample (cocktail inhibitor solution: 2.0 M Tris, 0.9 M Na-EDTA, 0.2 M Benzamidine, 92 *μ*M E-64, and 48 *μ*M Pepstatin; Sigma, St. Louis, Mich, USA). The plasma was stored at −80°C.

#### 2.2.2. Assay

**Figure 1:**
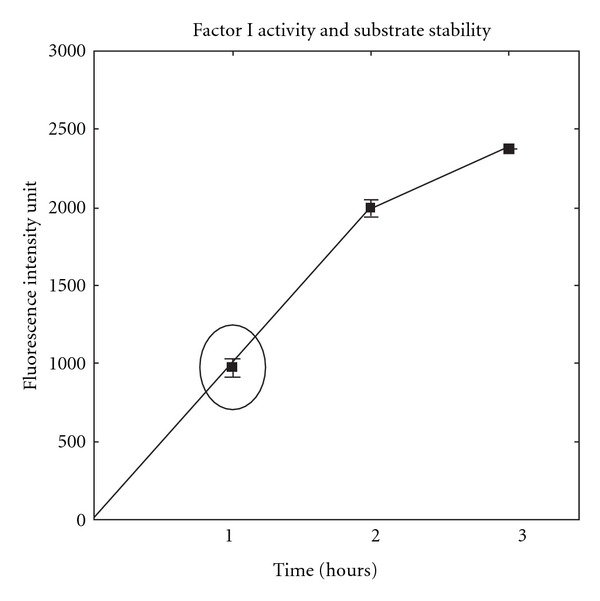
Fluorescence intensity of release of 7-amino-4-methyl coumarin as a function of plasma incubation period (**m** **e** **a** **n** ± **S** **D**; 1 h (974 ± 44.2), 2h (1995 ± 45.2) and 3 h (2374 ± 1.2)).

Methods based on the hydrolysis of fluorogenic substrates have previously been described by Tsiftoglou and Sim [36] and Gupta et al. [15]. The following assay procedure was found to be optimal for assaying fI activity in the plasma. 20 *μ*L of plasma was incubated with 80 *μ*L of buffer (100 mM phosphate buffer, pH 7.5, containing 1 mM EDTA, 1 mM DTT and 1 mM sodium azide) for 10 min at 37°C to reach thermal equilibrium. 100 *μ*L of substrate solution (200 *μ*M Boc-Asp(OBz)-Pro-Arg-7-amino-4-methylcoumarin; Bachem, Bubendorf, Switzerland) in 25 mM phosphate buffer, pH 7.4, was then added, and the mixture was incubated at 37°C for 60 min (see Figure 1). The reaction was inhibited by the addition of 1 mL of 1.5 M acetic acid, and the release of 7-amino-4-methylcoumarin was measured by spectrofluorometer (Hitachi-f 2000; *λ*_ex_: 360 nm; *λ*_em_: 440 nm; slit width: 2.5). All measurements were carried out randomized and in duplicate. Background fluorescence in the assay was monitored by the use of plasma in the absence of substrate and was subtracted from values obtained in the presence of substrate.

### 2.3. Data Analysis and Statistics

Plasma fI activity was log-normally distributed, and logarithmic values were, therefore, used when analysing differences between the ASD group and the control group. To adjust for age (dichotomized using the median value, 5 years) and gender, factorial ANOVA was used. A *P* value < 0.05 was considered statistically significant. Statistica 8.0 (StatSoft^©^, Tulsa, Okla, USA) was used. Intra- and interassay variability of the plasma fI activity was expressed as the standard error of a single determination (*S*_method_), using the formula first proposed by Dahlberg [37]

(1) $$S_{\text{method}}\;=\;\surd \left(\frac{\;{\sum d_{i}}^{2}}{\left(2n\right)}\right),$$

where *d*_*i*_ is the difference between the *i* : th paired measurement and *n* is the number of differences. The *S*_method_ was expressed as the coefficient of variation (%). 

## 3. Results

**Figure 2:**
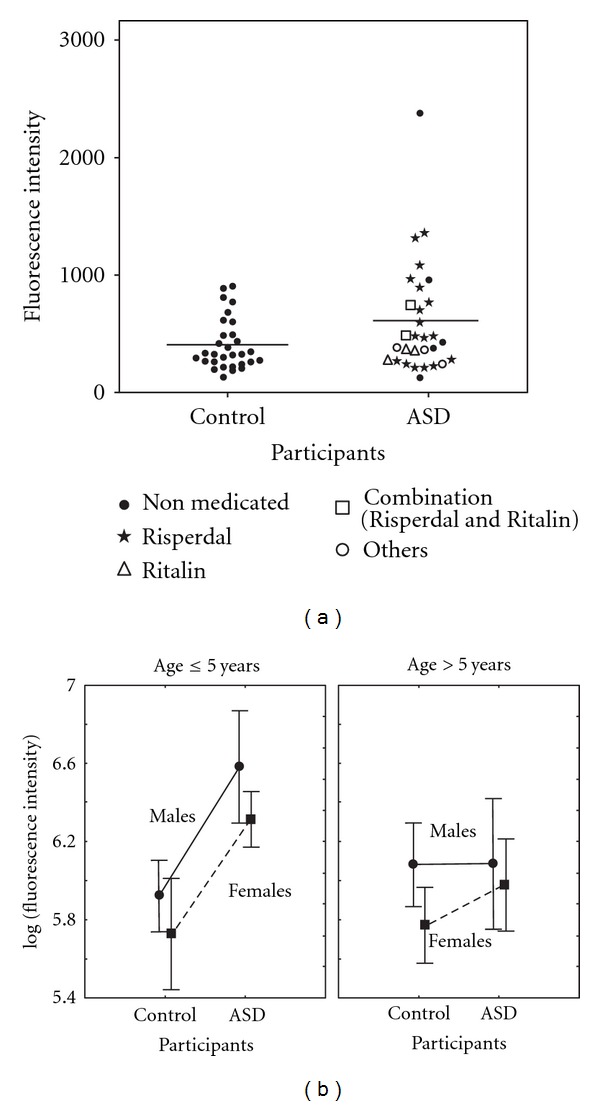
(a) Complement factor I activity in EDTA plasma from children with ASD (*n* = 30) and healthy control children (*n* = 30). A scatter plot of factor I activity for each individual is shown. Samples from ASD children who were not under medication at the time of the investigation, those under medication with Risperdal, Ritalin, a combination thereof, or other medications such as antipsychotics (thioridazine) or anticonvulsants (fenobarbital and sodium valproate) are shown. (b) Mean values and standard errors of the complement factor I activity in EDTA plasma are shown for the different age groups and genders for both children with ASD and the healthy control group. In the ASD group; age ≤ 5 years: males (*n* = 17), females (*n* = 4) and age > 5 years: males (*n* = 6), females (*n* = 3). In the healthy control group; age ≤ 5 years: males (*n* = 4), females (*n* = 10) and age > 5 years: males (*n* = 9), females (*n* = 7).

There was significantly higher activity of plasma fI in the children with ASD (geometric mean (95% confidence limit): 523 (154–1776) when compared with the control group: 361 (135–967; ANOVA *P* = 0.015, adjusted for age and gender; Figure 2). 

No statistically significant interactions were found with regard to gender and age, and no significant associations were found between fI activity and age or gender (ANOVA; *P* = 0.25 for gender and 0.42 for the two age groups, Figure 2). In the ASD group, some children with severe autism were under medication with Risperdal to reduce hyperactivity and violent behavior, and a few were under medication with Ritalin to improve attention (Table 1). It would have been ethically questionable to discontinue medication with the purpose of controlling the experimental design. We have correlated the data shown in Figure 2(a) with the type of medication the children in the ASD group were receiving. Although we did not see any clear correlation between medication and distribution for the scatter plot data, it cannot be excluded that some differences in the pattern may be influenced by medication, as has been previously discussed [22]. 

The values were statistically significantly higher in the children with ASD, and there was a weak association with gender. No statistically significant differences were found, however, between the age groups.

The methodological intra-assay error was small, 0.5%. The interassay methodological error was 13%. We found a significantly higher complement factor I enzyme activity in children with ASD compared to the control group of around the same age. This is, as far as we know, the first report regarding dysfunction of fI activity in children with ASD. Although not statistically significant, males tended to exhibit higher fI activity than females, and the difference between the control group and the ASD group was more convincing amongst the younger children, as shown in Figure 2. Due to fI's role as a regulating factor in the complement system pathway, an fI abnormality could play a role in the onset of ASD. A defect in this pathway makes the individual more vulnerable to various inflammations. Some reports [21, 24–26] provide increasing evidence of a connection between immunological abnormality and human disease. ASD numbers among the types of disorders that are associated with immune system abnormalities [28, 38]. Our results are consistent with a recent proteomic study of serum from ASD children, where a significant differential expression was shown for proteins related to the complement system [22].

## 4. Discussion

The aetiology of ASD is still largely unknown despite the fact that many factors such as genetic, environmental, immunological, and neurological aspects are thought to influence the development of ASD [39]. A dysregulated immune response has been reported among children with ASD. Protein products of immune activation, such as cytokines, can be linked to core features of ASD, such as difficulties to regulate affect, sleep, nutritional uptake, and can also affect behaviour and social communication [39]. This study highlights an elevated level of factor I that may contribute to a dysregulation of the immune response associated with the complement system. Recent research proposes an extensive communication between the immune and nervous systems, affecting both the development of the central nervous system [32] in the promotion of health as well as disease. Early damage during critical periods in neurodevelopment of the foetus has in a mouse model study been shown to affect cognitive development. It is reasonable to suggest that complement factor I might contribute to ASD, while changes in the complement system may predispose the mother or foetus to infections during development, possibly causing resultant abnormalities in brain development [22]. Also, the frequency of autoimmune diseases has been reported to be significantly higher in families with a child with ASD [40] than in families with children with typical development.

The pathophysiology of ASD is poorly understood. Children with ASD are prone to recurrent viral and bacterial inflammations. There are also some reports of immune system abnormalities in children with ASD [15–20]. An association between ASD and immune system abnormalities together with the vulnerability of the ASD group with regard to inflammatory processes may indicate an impaired mechanism in this system. It is known that phagocytosis is an important part of the body's defence mechanism. This mechanism requires an active complement system and a functioning C3b protein. C3b is degraded by fI, and abnormal fI activity might cause an abnormal and uncontrolled degradation of C3b protein, resulting in the loss of the phagocytosis function for this particular protein (a function which partly protects the body from invasion of foreign organisms). Altered levels of other serine protease activities, such as that of proline endopeptidase (PEP), have also been found in a group of children with ASD when compared to a control group [14]. The results of the present study, together with our previous findings on altered levels of PEP activity, may indicate a connection between the onset of ASD and serine protease dysfunction. The higher plasma fI activity observed in the male group as compared to that of the female group (Figure 2) is paralleled by a higher occurrence of ASD in male children. Also, the higher plasma fI activity in children younger than six years of age may indicate that the inflammatory process is more active in younger ages or that we may be dealing with two subgroups of children with ASD with different onsets of the disease.

## 5. Conclusions

The preliminary findings of this study together with our previous report [14] suggest that there may be an association between abnormal serine protease activity and the development of ASD. Further research is needed, however, to establish a possible role of serine proteases in the aetiology of ASD.

## Figures and Tables

**Table 1:** Age/y, gender, and medication of the participants.

Parameter		ASD (*n* = 30)	Controls (*n* = 30)	*P* value*
Age	Mean (SD)	4.8 (1.7)	6.1 (2.3)	
	Median (range)	4.5 (3–9)	6 (3–12)	0.033
	≤5 years	21	14	
	>5 years	9	16	0.115

Gender	Males	23	13	
	Females	7	17	0.017

Medication	No specific medication	8	30	—
	Risperdal alone or in combination	18	0	—
	Ritalin alone or in combination	4	0	—

*Difference between ASD and controls. Mann-Whitney *U*-test for age and Fisher's exact test for age category and gender.
